# From binary to synergy in precision mRNA therapeutics: Dual-microRNA-sensing mRNAs for modular logic control of protein expression

**DOI:** 10.1016/j.omtn.2025.102651

**Published:** 2025-08-13

**Authors:** Chermaine Tan, Sherry Aw

**Affiliations:** 1 Institute of Molecular and Cell Biology (IMCB), Agency for Science, Technology and Research (A∗STAR), 61 Biopolis Drive, Proteos, Singapore 138673, Republic of Singapore

## Main text

The programmability of RNA therapeutics and their demonstrated safe and efficacious use as vaccine medicines has heralded the beginning of an RNA medical revolution. To expand the treatable indications, the next generation of RNA therapeutics will require enhanced tunability and cell specificity, which can be achieved with tissue-specific targeted delivery, as well as improved RNA cargo design. In this study, Masaki et al.^1^ developed a programmable mRNA device for cell-specific protein production, which incorporates sensing of two cell-intrinsic microRNA (miRNA) signals into one mRNA. Their platform enables simultaneous selection for protein expression in the presence of one miRNA and suppression in the presence of another, allowing input of miRNA signals specific to both on-target and off-target cells, resulting in refinement of target cell-specific protein expression. This approach of engineering multi-component logic sensing into mRNA cargo, and their demonstration *in vivo*, is an exciting advance in the field as it looks toward next-generation, cell context-specific activation of mRNA therapies.

Over the last decade, great leaps have been made to address the formidable challenge of *in vivo* RNA delivery. Like any other class of therapeutics, toxicity and unwanted effects can result from off-target function of RNA therapies in non-target cells. To reduce these effects, improved targeted RNA delivery to specific organs, tissues or cell types has been achieved by altering the composition of lipid nanoparticles (LNPs),^2^ using cell-type-specific promoters on viral vectors,^3^ or by including cell-targeting ligands for cell-type-specific uptake.^4^ However, within a tissue type into which RNA can be delivered, achieving additional levels of control and precision remains challenging; for example, to distinguish between diseased and non-diseased cells of the same cell type, or to tune dosage based on cell context or fluctuating gene expression.

In light of this, the field has also employed synthetic *cis*-sequences to engineer control into the mRNA cargos themselves. The prevalence of single-cell RNA sequencing datasets and transcriptomic atlases of healthy and diseased tissues now allows routine identification of transcripts that are specifically expressed in cell types or disease states of interest. By programming into the mRNA cargo functional *cis*-elements that bind these cell-defining intracellular RNAs, translation can be specifically activated in target cells post-delivery. For example, binding of designer RNAs to specific transcripts create RNA modules that recruit adenosine deaminase acting on RNA to disable stop codons,^5^^,^^6^^,^^7^ or that regulate access to internal ribosomal entry sites.^8^ These innovative designer mRNAs demonstrate the ingenious ways that mRNA therapies can be designed to conditionally activate protein translation.

miRNAs bind to target mRNAs to downregulate protein expression, functioning as natural mRNA switches. Taking advantage of their programmability and the ubiquity of the miRNA machinery in mammalian cells, miRNA-binding sites have been introduced into exogenous mRNA cargos to control their translation in a cell-specific manner. For example, cell-specific miRNA-binding sites have been introduced into Cas9 mRNA to control Cas9 nuclease activity in a cell-type specific manner,^9^^,^^10^^,^^11^ into mRNAs coding for fluorescent proteins to enable cell purification,^12^ and into mRNAs coding for apoptotic proteins to target cancer cells.^13^

The aforementioned examples utilize encoded miRNA target sites to downregulate protein expression. Hirohide Saito’s group, one of the pioneers in leveraging cell-type-specific expression of miRNA to engineer switches to control translation, has termed this a microRNA “OFF-switch”^14^; their iteration incorporates a fully complementary binding site in the 5′-UTR for an off-target miRNA (*expressed in off-target cells*). Binding of a target miRNA to this site is predicted to recruit Argonaute 2 (AGO2)-mediated cleavage of the mRNA, separating the 5′ cap from the coding sequence. This suppresses translation, leading to decreased protein expression in these (off-target) cells. Following these studies, Fujita, Saito, and colleagues then ingeniously developed a microRNA-ON switch, which incorporates a binding site for a miRNA that is *expressed in on-target cells* after the polyA tail, followed by inhibitory “extra sequences.” Intracellular introduction of these mRNAs was found to result in lowered protein expression, while miRNA binding would lead to AGO2-mediated cleavage and removal of the inhibitory sequences, thereby relieving repression.^1^^,^^14^

In their recently published follow-up study,^1^ the authors improve on the efficacy of inhibitory “extra sequences” by investigating sequences of different composition and length. They found that six repeats of a UUUA motif was sufficient, removing need for addition of much longer sequences. Moreover, adding a U6 tract to the UUUA repeats further reduced leakiness of the ON switch. Using these newly identified sequences, the authors then combined ON and OFF logic into a single hybrid switch mRNA. This enabled selective expression of EGFP in induced pluripotent stem cells and not HeLa cells, with an ON/OFF expression ratio of up to 16-fold, which was superior to the individual ON and OFF switches. Finally, they evaluated the functionality of these switches *in vivo*. They found that the hybrid mRNA switch enabled selective expression in limb muscle but not in the liver following intramuscular injection, and also demonstrated selective expression in the liver but not the spleen post-intravenous injection.

Engineering RNA devices that sense an input, actuate a response, and generate an output has been explored for decades; however, achieving reliable control *in vivo* has been challenging. In this current study, miRNA sensing and actuation are mediated by endogenous, AGO2-dependent cleavage of the mRNA. The output, sustained or decreased protein expression, results from either the preservation or degradation of the mRNA, respectively. Hence, a single cleavage or no cleavage event is amplified into a robust OFF or ON protein output, underscoring the power of coupling endogenous mechanisms with engineered amplification.

As a field, engineering such devices still relies heavily on empirical screening during the early stages of development, with few universal rules or predictive design principles to guide the process. It would be interesting to explore why adding a U6-tract to different sequence repeats did not consistently reduce leaky expression. A better understanding of the mechanisms that underlie these differences could enhance our ability to rationally design such devices.

It is also important to note that the hybrid switch did not always outperform the individual ON or OFF switches; in the first *in vivo* experiment, the OFF switch alone achieved a ∼60-fold ON/OFF expression ratio, while the hybrid switch reached <20-fold, albeit still better than the ON switch alone, at ∼10-fold. This could arise from the decrease in translation overall elicited by the ON-switch, and reinforces the context-dependency inherent in RNA device engineering. While the second *in vivo* demonstration did show improved selective expression in the liver over the spleen using the hybrid mRNA switch, demonstration of selective activation in other non-hepatic tissues would be even more compelling.

These *in vivo* experiments demonstrated controlled expression of a fluorescent reporter protein; the next step would be application of this platform toward functional protein expression to drive phenotypic changes or therapeutic outcomes. This would allow investigation into whether the dynamic range of protein expression tuneable by miRNA input is sufficient to achieve therapeutically relevant effects. Characterization of the sensitivity and threshold parameters for miRNA selection and detection, including endogenous miRNA levels, the number of mRNA molecules delivered into each cell and the eventual functional output, will be important for selection of ideal use cases across biological or disease conditions.

This study demonstrates a strategy of progressive advancement from single-input, miRNA-responsive translational switches to modular, dual-input RNA-only circuits capable of precise therapeutic logic *in vitro* and *in vivo*. It lays the groundwork to explore and further layer other switches and inputs for a next generation of programmable mRNA control for precise, cell-specific protein expression.

## Declaration of interests

The authors are inventors on patents related to RNA-sensing technologies.
